# Spatiotemporal characteristics and meteorological determinants of hand, foot and mouth disease in Shaanxi Province, China: a county-level analysis

**DOI:** 10.1186/s12889-021-10385-9

**Published:** 2021-02-17

**Authors:** Li Ding, Ning Zhang, Bin Zhu, Jinlin Liu, Xue Wang, Feng Liu, Ying Mao

**Affiliations:** 1 School of Humanities and Social Science, Xi’an Jiaotong University, Xi’an, China; 2 Health Commission of Xi’an, Xi’an, China; 3 School of Public Policy and Administration, Xi’an Jiaotong University, Xi’an, China; 4 Research Center for the Belt and Road Health Policy and Health Technology Assessment, Xi’an Jiaotong University, Xi’an, China; 5 Department of Public Policy, City University of Hong Kong, Hong Kong, China; 6 Water H. Shorenstein Asia-Pacific Research Center, Stanford University, Stanford, CA USA; 7 The Second Affiliated Hospital of Xi’an Jiaotong University, Xi’an, China; 8 Shaanxi Provincial Centre of Disease Control and Prevention, Xi’an, China

## Abstract

**Background:**

Hand, foot and mouth disease (HFMD) is one of the common intestinal infectious diseases worldwide and has caused huge economic and disease burdens in many countries. The average annual incidence rate of HFMD was 11.66% in Shaanxi during the time span from 2009 to 2018. There are distinct differences within Shaanxi, as it is a special region that crosses three temperature zones. Hence, in this study, a spatiotemporal analysis of Shaanxi was performed to reveal the characteristics of the distribution of HFMD and to explore the meteorological determinants of HFMD.

**Methods:**

The county-level and municipal data from Shaanxi Province from 2009 to 2018 were applied to research the spatiotemporal characteristics of HFMD and its meteorological determinants. Time series and spatial autocorrelation analyses were applied to assess the spatiotemporal characteristics of HFMD. This study used spatial econometric panel models to explore the relationship between HFMD and meteorological factors based on the data of 107 counties and 10 municipalities.

**Results:**

The incidence rate of HFMD displayed no variable trend throughout the whole research period. A high incidence rate of HFMD was observed from June to September, corresponding to a time when the climate is characterized by heavy rain, high temperature, and high humidity. The high-incidence areas were mainly located in the central region in Shaanxi, whereas the low-incidence spots were mainly found in Northern Shaanxi. Regarding the meteorological factors analysed in this study, in general, the incidence rate of HFMD in specific regions was positively associated with the rainfall, temperature and humidity.

**Conclusion:**

These results could be applied by the government and the general public to take effective measures to prevent disease. Region-targeted policies could be enacted and implemented in the future according to specific situations in different areas and the relevant meteorological determinants. Additionally, meteorological conditions normally extend to a wide-ranging region; thus, cooperation among surrounding regions is necessary.

**Supplementary Information:**

The online version contains supplementary material available at 10.1186/s12889-021-10385-9.

## Background

Hand, foot and mouth disease (HFMD) is one of the common infectious diseases caused by enteroviruses and can normally cause fever and rashes or ulcers on the hands, feet, mouth and other parts. Some patients can experience encephalitis, acute flaccid paralysis, respiratory infections, and myocarditis [1]. The epidemiological characteristics of HFMD are as follows: the population is generally susceptible; however, the disease mostly occurs in infants and young children under 5 years old [2, 3]. Children and adults often do not experience symptoms after infection, but they can spread the virus [4]. The virus is widespread and can have outbursts in all four seasons, especially in summer. When the disease breaks out, the young are often more susceptible to infection than adults.

HFMD has caused huge economic and disease burdens worldwide [5]. In 2011, the World Health Organization (WHO) issued a guide to clinical management and public health response for HFMD to tackle the disease [6]. The guide includes the epidemiology, virology, laboratory diagnosis, and pathogenesis of EV71 infection, along with prevention and control measurements. Globally, outbreaks of HFMD have been found in America [7], Spain [8], Brazil [9], and Finland [10]. However, the western Pacific region is most affected, namely, Japan [11], Thailand [12], Singapore [13], and Korea [14]. In China, the Chinese Center for Disease Control and Prevention (CDC) included HFMD as a Class C reported notifiable infectious disease. In 2008, 31 provinces in China reported 488,955 cases of HFMD, 1165 were severe cases, and 126 died. The incidence and mortality rates were 37.01/100,000 and 0.03%, respectively [15]. Based on calculation, in 2018, 2,353,310 cases and 35 deaths due to HFMD were reported in China. The incidence and mortality rates were 169.41 /100,000 and 0.0025/100,000, respectively. The average annual growth rate of HFMD incidence is 16.43%. The incidence rate of HFMD in Shaanxi province from 2009 to 2018 was 140.04/100,000, which is higher than the national reported incidence rate in the same period. Moreover, there are distinct differences within Shaanxi, as it is a special region that crosses three temperature zones. Hence, in this study, a spatial-temporal analysis of Shaanxi was performed to reveal the characteristics of the distribution of HFMD and to explore the meteorological determinants of HFMD.

Previous studies have been conducted to analyse the spatial-temporal characteristics of HFMD and the explanatory determinants. However, few studies researched the spatial-temporal situation in Shaanxi, China [16, 17]. Furthermore, different determinants have been explored in several studies, though no spatial determinants have been researched, meaning that all determinants included were derived from the local region rather than other surrounding regions. From the perspective of characteristics, Mao used seasonal decomposition, spatial autocorrelation and space scanning methods to analyse the all the intestinal infectious diseases in China. HFMD displayed a distinct increasing trend. The high-risk areas for HFMD were located in the Beijing-Tianjin-Tangshan (BTT) region and south China [18]. Hassel used large-panel datasets and a Bayesian phylogenetic approach to compare the molecular epidemiology and geographical spread patterns in Europe [19]. Bian researched the different pathogens of HFMD worldwide [20]. Regarding the determinants, the researchers mainly focused on individual, socio-ecological, geographical and meteorological factors. Lee used a general additive model (GAM) to explore the relationship between HFMD and geographical factors, as well as meteorological factors in East Asia [21]. Koh conducted a systematic review of the epidemiology of hand, foot and mouth disease in Asia and found that risk factors for HFMD included hygiene, age, gender and social contacts [22]. The research of Hao found that the incidence rate of HFMD was significantly associated with average temperature, relative humidity, vapor pressure, and wind speed. As can be seen from the above studies, spatial and temporal characteristics-related research has been performed in some regions of China using various methods such as time series, seasonal decomposition spatial autocorrelation and space scanning; however, there has been no specific study focusing on Shaanxi province. In addition, almost all the research analysed the meteorological factors related to HFMD. However, there is limited research discussing meteorological factors and the spatial dynamic patterns of HFMD. Determining the meteorological factors would essential for studying the relationship with the spatial dynamic patterns of HFMD.

In general, this study analysed the general distribution of HFMD from 2009 to 2018 using county-level and municipal data in Shaanxi, China. Then, the temporal and spatial characteristics of HFMD were evaluated. We then used a spatial econometric panel model to explore the spatial relationships between HFMD and related factors.

## Methods

### Study area

**Fig. 1:**
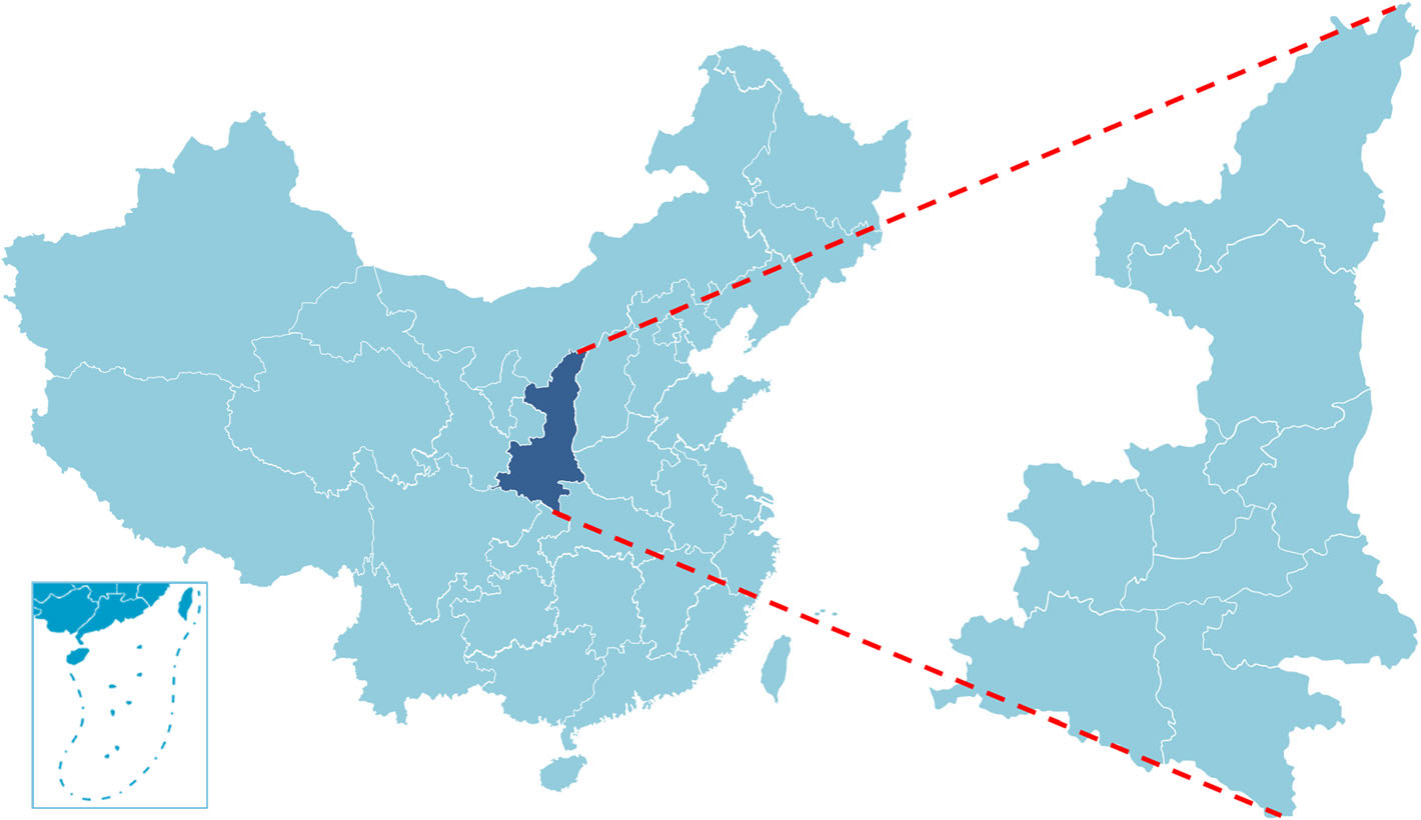
The map of Shaanxi province, China (made through Microsoft PowerPoint, source of map: http://www.geodata.gov.cn/web/geo/index.html, under license without need for permission). *This figure was made by ourselves through Microsoft PowerPoint (Version 2013, Microsoft Crop, Redmond, WA, USA). The source of map was a public database, National Nature Resources and Geospatial basic information database of PRC (http://www.geodata.gov.cn/web/geo/index.html). This maps were under license without need for permission

Shaanxi is located in central China between 105°29′ E-111°15′ E and 31°42′ N—39°35′ N, with the capital of Xi’an. Shaanxi has 10 municipal administrative divisions and 107 county administrative divisions. The terrain of Shaanxi Province is high in the north and south and low in the middle and is composed of plateaus, mountains, plains and basins. It covers the two major rivers: the Yellow River and the Yangtze River. Shaanxi has three climate types, namely moderate temperate monsoon climate, warm temperate monsoon climate and subtropical monsoon climate. In 2018, Shaanxi had a population of 38.844 million, with a GDP of 24.43832 billion yuan. Figure 1 displays a map of Shaanxi province.

### Data source and measurement of variables

#### Data source

The county-level and municipal data of Shaanxi province from 2009 to 2018 were applied to research the spatio-temporal characteristics of HFMD and its meteorological determinants. The incidence rate of HFMD was obtained from the Shaanxi Provincial Center for Disease Control and Prevention (SXCDC). The meteorological data were obtained from the China Meteorological Data Service Center (CMDC), which is a subsidiary of the China Meteorological Administration (CMA). The monthly incidence rates of HFMD in all counties and meteorological data (Appendix 1) from all municipal cities were collected from 2009 to 2018. The descriptive statistics of HFMD are shown at the municipal level as a limitation of length, while temporal and spatial autocorrelation analyses were conducted using county-level data. Finally, the spatial econometric panel model used monthly municipal data. Table 1 displays the variables and data sources.

**Table 1:** Variables and data sources

Variables	Research subjects	Research period	Date resources
HFMD	10 municipal cities/107 counties	2009.01–2018.12	SXCDC
Waterfall	10 municipal cities	2009.01–2018.12	CMDC
Temperature	10 municipal cities	2009.01–2018.12	CMDC
Humidity	10 municipal cities	2009.01–2018.12	CMDC

#### Measurement of variables

This study took the incidence rate of HFMD as a dependent variable and the meteorological variables, namely rainfall, temperature and humidity, as independent variables. Specifically, the incidence rates of HFMD is calculated through reported cases and potential population, which was normally obtained from SXCDC directly. The meteorological variables were obtained from CMDC through meteorological stations. Table 2 displays the variable measurements and descriptions.

**Table 2:** Variables measurements and description

Variables type	Variable name	Measurement	Description
Dependent variables	HFMD	The incidence rate of HFMD	Number of new cases of HFMD in population during a certain period of time / Number of people exposed during the same period
Independent variables	Waterfall	Average monthly rainfall	Add up the average daily rainfall and divide by the number of days in the month
	Temperature	Monthly average temperature	Add up the average daily temperature and divide by the number of days in the month
	Humidity	Monthly average relative humidity	Add up the average daily humidity and divide by the number of days in the month

### Temporal analysis method

Time series were applied via a moving average method and are displayed through the plot. The moving average method was applied to smooth out short-term fluctuations and highlight longer-term trends or cycles [23]. In this article, the raw monthly incidence rate and estimated moving averages of 4 months (seasonally) and 12 months (yearly) are displayed in the plot.

### Spatial analysis method

#### Spatial autocorrelation analysis

There are two main laws in geography. The first one was proposed by Waldo Tobler: “Everything is associated with others, and close things are more related compared with distant things” [24]. The first law demonstrates the relationship between distance and association. Michael Goodchild came up with the second law, the 1aw of spatial heterogeneity: “The separation of space accounts for the difference between regions, namely, heterogeneity, including spatial local heterogeneity and spatial stratified heterogeneity” [25]. The second law illustrates that the specific values of units were different from the surrounding regions, which could be regarded as hot or cold spots. Based on the laws of geography, spatial autocorrelation analysis was formulated to reveal the spatial dependence and hierarchical spatial enumeration. Appendix 2 provides a demonstration of different types of spatial cluster situations. Each circle represents the variables in specific units, and the circles are associated with each other. The red circles represent indicators with higher values, while the blue circles denote the lower values. The left graph, demonstrating the positive spatial autocorrelation, shows the pattern of clusters with similar values, namely, the red circles tend to be near to each other, and the blue circles surround each other. There is no spatial autocorrelation in the middle graph due to the random distribution of high and low values. Negative spatial autocorrelation is found in the right graph, which means that the high values are surrounded by the low values.

The Moran’s I is one of the most commonly used indicators considering spatial autocorrelation analysis, which consists of global and local Moran’s I. Global Moran’s I is a reflection of the first law, measuring the spatial dependence of the whole research region, while as a transformation of the second law, the local Moran’s I reflects the regional differences. In our study, the global and local Moran’s I findings reveal the whole-level spatial distribution characteristics of the study region and specific cluster regions in the research area, respectively [26].

In this study, the value of global Moran’s I, ranging from − 1 to 1, reflects the overall spatial distribution of HFMD in Shaanxi province. When the index is near 1, a positive spatial autocorrelation is detected [27, 28]. The counties with high incidence rates of HFMD tend to cluster. A zero means that there is no spatial autocorrelation of HFMD, illustrating high and low values scattered randomly in Shaanxi. When the values are distributed around − 1, a negative spatial autocorrelation is observed, indicating that counties with high and low values border each other. The equation of global Moran’s I is as follows:

$$ Global\ {Moran}^{\prime }s\ I=\frac{n{\sum}_{i=1}^n{\sum}_{j=1}^n{W}_{ij}\left({X}_i-\overline{X}\right)\left({X}_j-\overline{X}\right)}{\sum_{i=1}^n{\sum}_{j=1}^n{W}_{ij}{\left({X}_i-\overline{X}\right)}^2} $$

Where *X*_*i*_ is the incidence rate of HMFD in county i and j. The $\overline{X}$ is the mean value of the incidence rate of HFMD in Shaanxi. The difference between the mean and absolute values of incidence rate is crucial in determining the positive or negative effects. *n* is the number of all the counties in Shaanxi. *W*_*ij*_ is an important tool in spatial modelling, as it quantifies the spatial dependence between observations, which is normally expressed as an n × n non-negative matrix W:

$$ {W}_{ij}=\left[\begin{array}{ccc}{w}_{11}& \cdots & {w}_{1n}\\ {}\vdots & \ddots & \vdots \\ {}{w}_{n1}& \cdots & {w}_{nn}\end{array}\right] $$

Where n is the number of spatial units; *W*_*ij*_ represents the spatial dependency relationship between region i and region j. The larger the weight value, the stronger the spatial dependency between regions. The spatial weight matrix was constructed based on a contiguity relationship. Therefore, the value on the main diagonal of the matrix is zero, which means that each area is not adjacent to itself, namely, *W*_*ij*_ = 0. At the same time, if areas i and j are adjacent, then *W*_*ij*_ = *W*_*ji*_. The spatial weight matrix is symmetrical.

Regarding the local Moran’s I, a positive value of the index represents the similarity of region, which means that the regions with high or low incidence rates of HFMD cluster within the same category, while a negative value indicates the opposite, that is, the counties with high incidence rates tended to be near regions with low incidence rates. Based on the value and the significance level, the clusters could be classified as four types, namely, High-high (HH, the regions with high incidence rates are surrounded with other high incidence rate regions), High-low (HL), Low-high (LH), and Low-low (LL). The equation of local Moran’s I is as follows [26]:

$$ Local\ {Moran}^{\prime }s\ I=\frac{x_i-\overline{x}}{m_0}{\sum}_j{W}_{ij}\left({X}_j-\overline{X}\right){m}_0={\sum}_i{\left({X}_i-\overline{X}\right)}^2/n. $$

Where *m*_0_ is a constant across all county-units; the explanation of other parameters is the same as with the global Moral’s I. To further demonstrate the statistically significant level of the incidence rate of HFMD, a map displaying the counties whose local Moran’s I has significant results is presented. The map is also known as a LISA map.

#### Spatial econometric panel model

In this study, the Spatial Lag Panel Model (SLPM), Spatial Error Panel Model (SEPM), and Spatial Durbin Panel Model (SDPM) were introduced to reveal the relationship between the HMFD and meteorological factors based on the following model derived from the measurement of variables [29–32]. The logarithm of the variable would not change the nature and correlation of the data, but it would compress the scale of the variable. After taking the logarithm of the variables, the data was more stable, and the collinearity and heteroscedasticity of the model were also weakened. In this article, the logarithm of waterfall played an important in weakening the heteroscedasticity. Besides, the temperature had negative number and the unit of humidity is percentage, which is not suitable for logarithm change, so the final model was as follows:

$$ \ln {(HFMD)}_{it}=\alpha +{\beta}_1\ln {(waterfall)}_{it}+{\beta}_2{temperature}_{it}+{\beta}_3{humidity}_{it}+{\upvarepsilon}_{ij}\ i=1,2\dots 107;t=2009,2010\dots 2018 $$

Where the *i* represents the 107 county-units (*i* = 1, 2…107); *t* means the time variable (*t* = 2009, 2010…2018); *α* denotes the constant term and *ε*_*ij*_ represents the error term. The SLPM is used to analyse the influence of dependent variables from the neighbouring counties by adding the spatial lag term of the dependent variable into the independent variable. The spatial dependence can be reflected as an error term, namely, missing variables in the model have a spatial correlation with HMFD, or unobservable random variables have spatial correlations with HMFD. The SEPM is applied in such circumstances. The SDPM is useful in reflecting the influence on specific regions from surrounding regions. However, although the SDPM can reveal the relationship between dependent and independent variables inside and outside the local region, the coefficients of SDPM cannot be directly explained, as the effects due to the derivative of *y* correspondence to *x* usually do not equal *β*_*k*_. Hence, the effects of the coefficient can be decomposed into direct and spill-over effects.

After understanding the functions of all the spatial econometric panel models, a standard model selection strategy is established. The procedures can be divided into four steps. In the first step, the Moran’s I or LM test is introduced to examine the spatial autocorrelation, namely, the availability of conducting spatial analysis methods. In the second step, the Wald test and the LR test are used to choose the SLPM, SEPM or SPDM. In the third step, the Hausman test is applied to determine whether a fixed effect model or a random effect model should be used. If a fixed effect model is used, the last step is introduced to determine the application of fixed effects (time, individual or both). If it is fixed effect model, the last step were introduced to determine individual fixed effects (controlling the “space-specific, time-invariant” variables, which are excluded from the model) or time effects (controlling the “time-specific, space-invariant” variables, which are excluded from the model) or both fixed effects (controlling the above two), and it would be chosen according to the sample size and time.

### Software tools

The time series analysis used Microsoft Excel (Version 2013, Microsoft Crop, Redmond, WA, USA) for visualization. The spatial autocorrelation analysis and the spatial weight matrix were analysed by GeoDa (Version 1.8.61, the University of Chicago, Chicago, IL, USA). STATA 15.0 (Version 15.0, StataCorp, College Station, TX, USA) was employed to calculate the spatial panel models. Finally, ArcGIS (Version 10.0, ESRI Inc., Redlands, CA, USA) was used to visualize the results.

## Results

### The prevalence of HFMD

We listed the morbidity of HFMD reported in each municipal unit in Shaanxi from 2009 to 2018. Table 3 provides a descriptive analysis of HFMD, containing the average, maximum and minimum values of morbidity at the municipal level from 2009 to 2018. The municipal administrative divisions were classified as northern, central and southern Shaanxi according to the geographical difference.

**Table 3:** Descriptive statistics of the variables

Variable	Obs	Mean	Std. Dev.	Min.	Max.	Units
HFMD	1200	9.449	12.335	0	100.0865	/10,000 person
waterfall	1200	56.523	60.215	0	367.05	mm
temperature	1200	12.211	9.358	−12.175	30.5	°C
humidity	1200	65.642	12.512	29	91	%

As shown in the analysis of the descriptive summary provided in Table 4, Central Shaanxi had the highest incidence rate compared with the northern and southern units, with an average of 13.87 per 100,000 individuals in 2018, while the morbidity averages in the northern and southern units were 12.55 per 100,000 individuals and 6.68 per 100,000 individuals, respectively. At the same time, the incidence in the southern region was relatively high in comparison with the northern one. The central region showed peak of morbidity in 2015, compared to peaks in 2018 and 2014 for the northern and southern units, respectively. When considering the municipal cities, Xi’an, the capital, had the most severe situation, with an average incidence of 20.92 per 100,000 individuals during the research period. Weinan, a city in central Shaanxi, experienced a relatively serious situation in addition to Xi’an. Yan’an and Yulin, cities in northern Shaanxi, were regarded as the least affected areas.

**Table 4:** The total incidence rates of HFMD in Shaanxi from 2009 to 2018 (1/100,000)

Region		2009	2010	2011	2012	2013	2014	2015	2016	2017	2018	Average
Xi’an	IN	12.43	27.12	12.20	24.83	19.42	24.50	27.97	21.00	14.07	25.65	20.92
	Min.	3.80	8.76	2.22	9.35	7.07	13.01	16.27	9.99	5.97	8.42	8.49
	Max.	27.93	55.30	20.86	41.83	36.16	42.94	45.68	38.46	26.81	46.47	38.24
Baoji	IN	3.91	8.61	5.20	9.02	7.34	11.18	8.65	5.77	3.90	13.66	7.72
	Min.	0.63	0.77	0.37	4.39	1.18	5.81	2.63	2.37	1.46	4.42	2.40
	Max.	12.26	18.34	17.55	28.84	20.00	25.91	14.09	16.81	8.09	31.32	19.32
Tongchuan	IN	4.52	5.71	3.75	8.63	6.87	8.90	8.42	5.61	4.27	7.84	6.45
	Min.	1.61	3.48	2.41	5.52	4.73	4.78	5.75	3.16	3.57	4.86	3.99
	Max.	8.45	9.75	4.30	12.00	9.93	12.47	11.04	8.65	5.82	13.75	9.62
Weinan	IN	9.04	14.94	12.52	12.10	14.22	20.28	18.81	13.38	9.25	10.35	13.49
	Min.	1.54	3.56	2.32	4.24	3.58	8.25	5.48	2.08	3.77	2.44	3.73
	Max.	29.28	27.02	26.00	22.63	37.94	37.29	41.68	25.75	15.30	18.05	28.09
Xianyang	IN	6.61	9.23	4.41	14.99	10.65	15.90	12.79	10.50	6.02	11.86	10.30
	Min.	1.79	2.04	0.49	3.40	1.82	5.53	5.68	4.38	2.79	4.53	3.25
	Max.	19.20	19.02	13.07	32.67	24.57	29.53	27.35	20.44	10.44	18.12	21.44
**Central Shaanxi**	IN	7.30	13.12	7.62	13.91	11.70	16.15	15.33	11.25	7.50	13.87	11.78
Shangluo	IN	2.91	7.47	4.63	9.95	7.99	9.09	11.02	6.28	7.14	9.84	7.63
	Min.	1.20	3.28	1.47	2.84	2.85	1.55	2.11	1.56	1.51	4.64	2.30
	Max.	6.43	13.88	8.88	20.28	13.16	20.93	24.35	11.94	15.15	21.36	15.64
Ankang	IN	3.78	7.83	3.16	7.69	10.79	9.47	11.82	14.61	7.32	16.58	9.31
	Min.	0.07	0.70	0.11	1.08	0.78	1.05	0.49	4.13	4.07	4.04	1.65
	Max.	12.05	11.25	13.77	18.53	69.52	21.04	19.48	31.35	16.67	21.51	23.52
Hanzhong	IN	1.78	12.43	2.89	8.90	11.54	12.32	7.76	8.90	7.02	11.22	8.48
	Min.	0.00	0.72	0.30	2.45	2.74	3.88	1.53	2.72	0.82	4.67	1.98
	Max.	5.34	21.40	7.10	15.15	22.48	24.13	14.51	21.83	11.59	18.67	16.22
**Southern Shaanxi**	IN	2.82	9.24	3.56	8.85	10.11	10.29	10.20	9.93	7.16	12.55	8.47
Yan’an	IN	2.85	2.86	2.96	1.15	3.70	10.04	4.85	4.13	10.07	5.72	4.83
	Min.	0.11	0.14	0.21	0.20	0.23	1.02	0.49	0.46	1.09	0.34	0.43
	Max.	11.22	17.40	17.52	8.98	16.22	43.32	25.21	21.29	32.25	22.08	21.55
Yulin	IN	4.01	4.87	4.26	4.08	4.27	6.08	9.36	4.29	4.82	7.64	5.37
	Min.	0.27	0.62	0.43	0.75	0.86	1.10	0.99	0.50	0.84	0.93	0.73
	Max.	17.49	12.88	19.20	12.08	18.22	14.84	45.07	8.82	8.90	17.63	17.51
**Northern Shaanxi**	IN	3.43	3.87	3.61	2.62	3.99	8.06	7.11	4.21	7.45	6.68	5.10
**Sum**	IN	6.49	12.94	7.07	12.87	11.68	15.55	15.17	11.62	8.68	14.58	11.67

Note: *IN* Incidence rate, *Min.* Minimum, *Max.* Maximum

### Temporal analysis

**Fig. 2:**
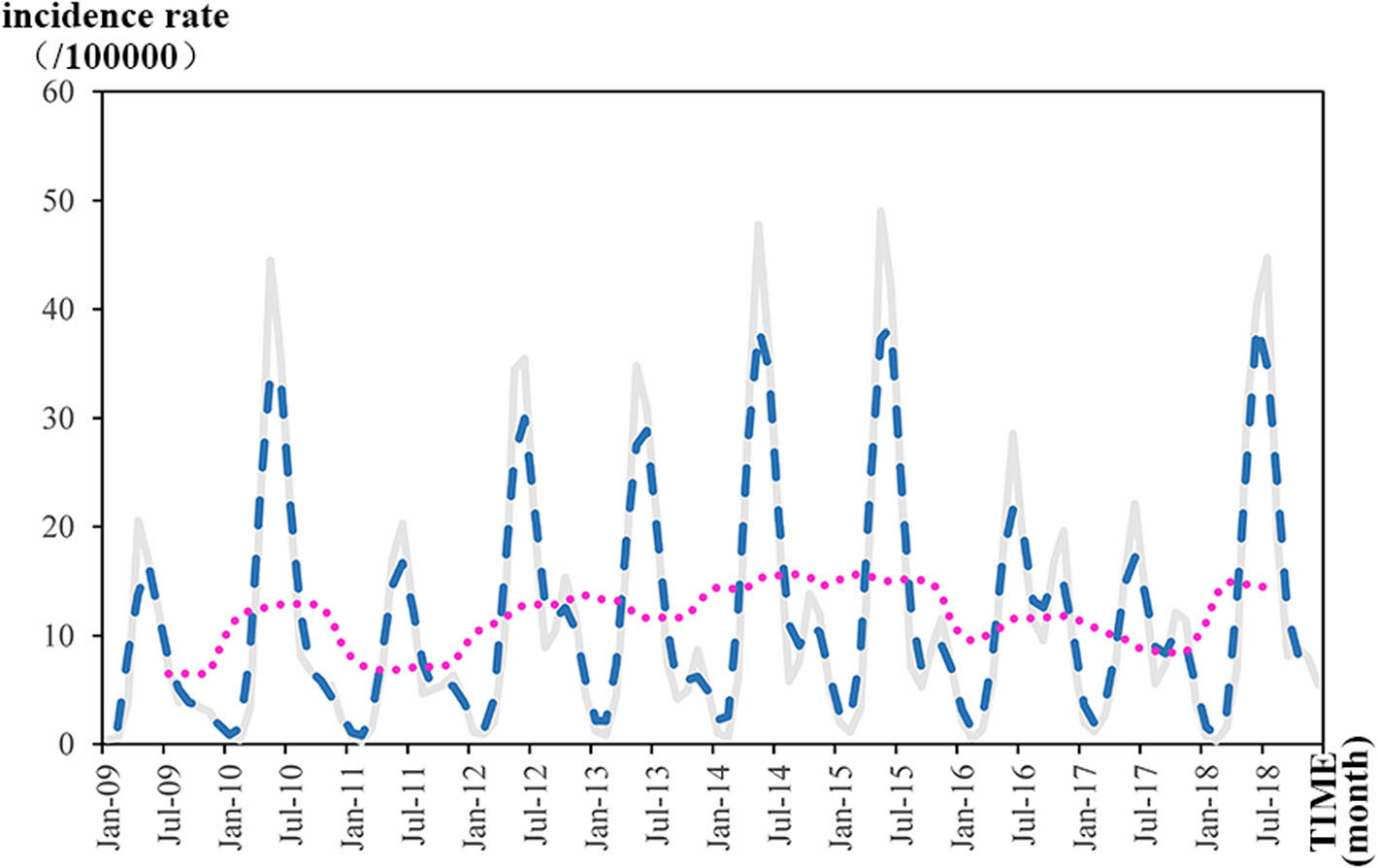
Time series analysis plots (made through Microsoft Excel). Note: The incidence rate (gray lines) were calculated through the incidence rate and population. Moving average data of 3 months (blue lines) and 12 months (pink lines) were shown. *This figure was made by ourselves through Microsoft Excel (Version 2013, Microsoft Crop, Redmond, WA, USA)

Time series analysis plots are shown in Fig. 2. In the trend analysis, there was no distinct trend during the research period. Relatively high incidence rates were witnessed in 2010, 2012, 2013, 2014, 2015 and 2018. In contrast, relatively low incidence rates were found in 2009, 2011, 2016 and 2017. In the seasonal analysis, the high-incidence seasons of HFMD in the study period were summer and autumn according to the different peaks throughout the years.

### Spatial autocorrelation analysis

The spatial autocorrelation analysis was analysed from two perspectives: global and local spatial autocorrelation. The function of global spatial autocorrelation was to detect whether the incidence rate of Shaanxi tended to cluster from 2009 to 2018. The function of local spatial autocorrelation was to differentiate the type of cluster.

#### Global spatial autocorrelation

Table 5 illustrates the Moran’s I and the significance level of HFMD from 2009 to 2018. In consideration of the significance results, all the Moran’s I values of HFMD were significant. Table 5 also reports the significance levels of all counties. Considering 2009 as an example, the number of counties with a 0.001 significance level was 6, while 5 and 15 counties corresponded to significance levels of 0.01 and 0.05, respectively. Considering the global Moran’s I, the index of HFMD ranged from 0.177130 to 0.514433 throughout the 10 years, without a distinct increasing or decreasing trend. In general, there was an evident spatial cluster of HFMD during the study period, allowing subsequent analysis of the local spatial autocorrelation.

**Table 5:** Global spatial autocorrelation analysis and significance test results

Year	Moran’s I	*p*-value	p-0.05	P-0.01	p-0.001	p-0.0001
2009	0.270299**	0.00170	15	5	6	0
2010	0.514433***	0.00001	8	9	7	5
2011	0.386398***	0.00001	13	11	3	1
2012	0.500738***	0.00001	7	11	11	4
2013	0.177130**	0.00476	20	8	3	2
2014	0.271359***	0.00002	21	11	2	0
2015	0.328566***	0.00001	20	9	5	0
2016	0.311582***	0.00002	12	12	8	2
2017	0.237429***	0.00054	19	9	2	0
2018	0.352848***	0.00001	14	11	6	3

Note: *a 10% level of statistical significance; **a 5% level of statistical significance; ***a 1% level of statistical significance

#### Local spatial autocorrelation

**Fig. 3:**
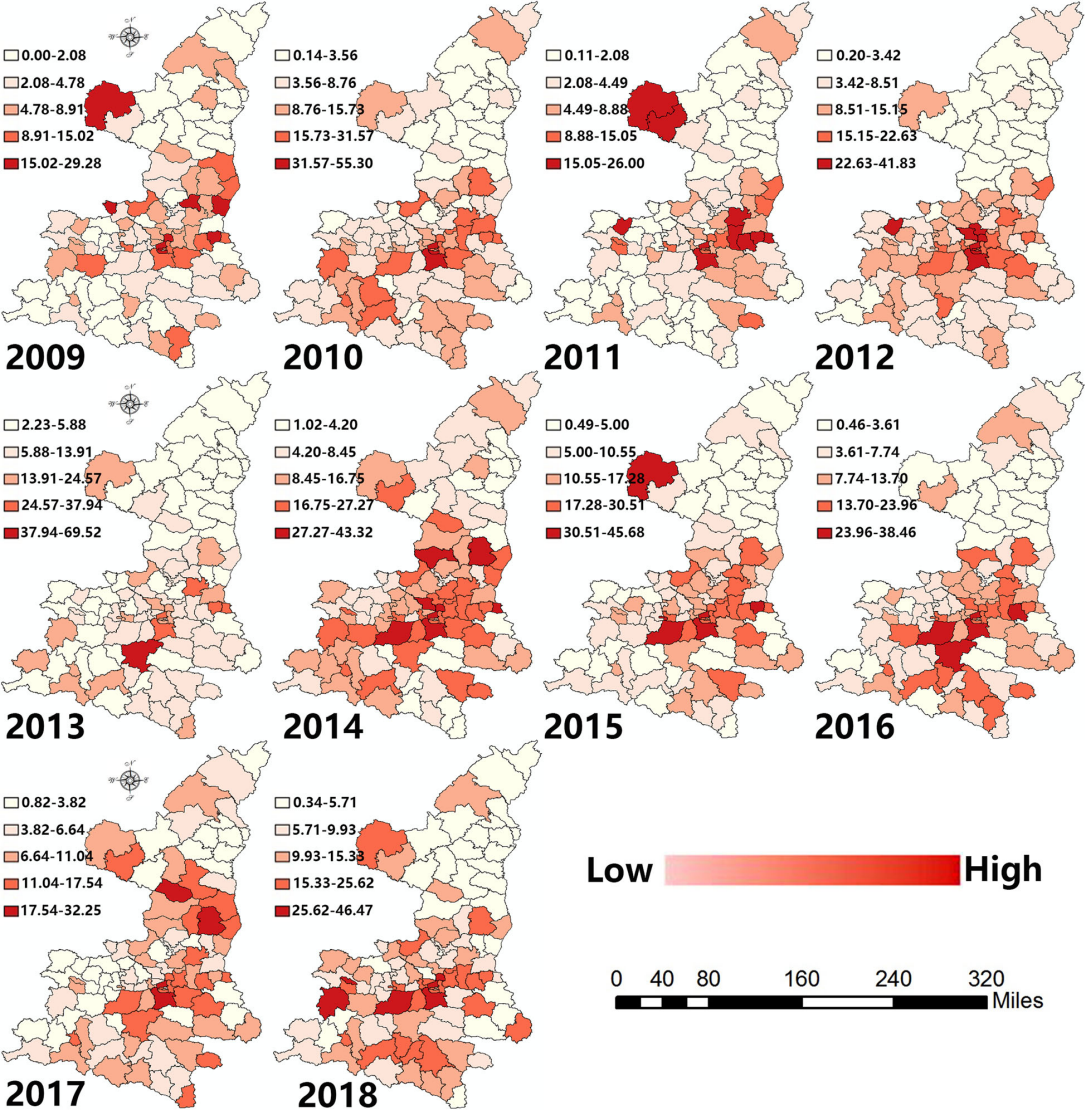
Map showing the hierarchy of the incidence rates for HFMD (made through GeoDa and ArcGIS, source of shape files: http://www.geodata.gov.cn/web/geo/index.html, under license without need for permission). *This figure was made by ourselves through GeoDa (Version 1.8.61, the University of Chicago, Chicago, IL, USA) and ArcGIS (Version 10.0, ESRI Inc., Redlands, CA, USA). The source of shape files was a public database, National Nature Resources and Geospatial basic information database of PRC (http://www.geodata.gov.cn/web/geo/index. html). Those shape files were under license without need for permission

Figure 3 displays the incidence rate of HFMD in all counties of Shaanxi from 2009 to 2018. The incidence rates were classified into five categories based on the minimum and maximum values. The classification method was natural segmentation using ArcGIS. The deeper the red colour, the higher the incidence rate. Considering morbidity in 2009 as an example, the minimum and maximum values were 0.00 per 100,000 individuals and 29.28 per 100,000 individuals, respectively, with categories divided into 0.00–2.08, 2.08–4.78, 4.78–8.91, 8.91–15.02, and 15.02–29.28. We found that central Shaanxi and counties in the northwest had the highest detected incidence rates. It should also be noted that the classifications of HFMD were different due to the high variation throughout the research period. Using the same classification method would lead to no differences detected between counties in some years. In general, the hierarchical maps only illustrate the relative relationship within a single year. Appendix 3 displays the map with same legend for different years.

**Fig. 4:**
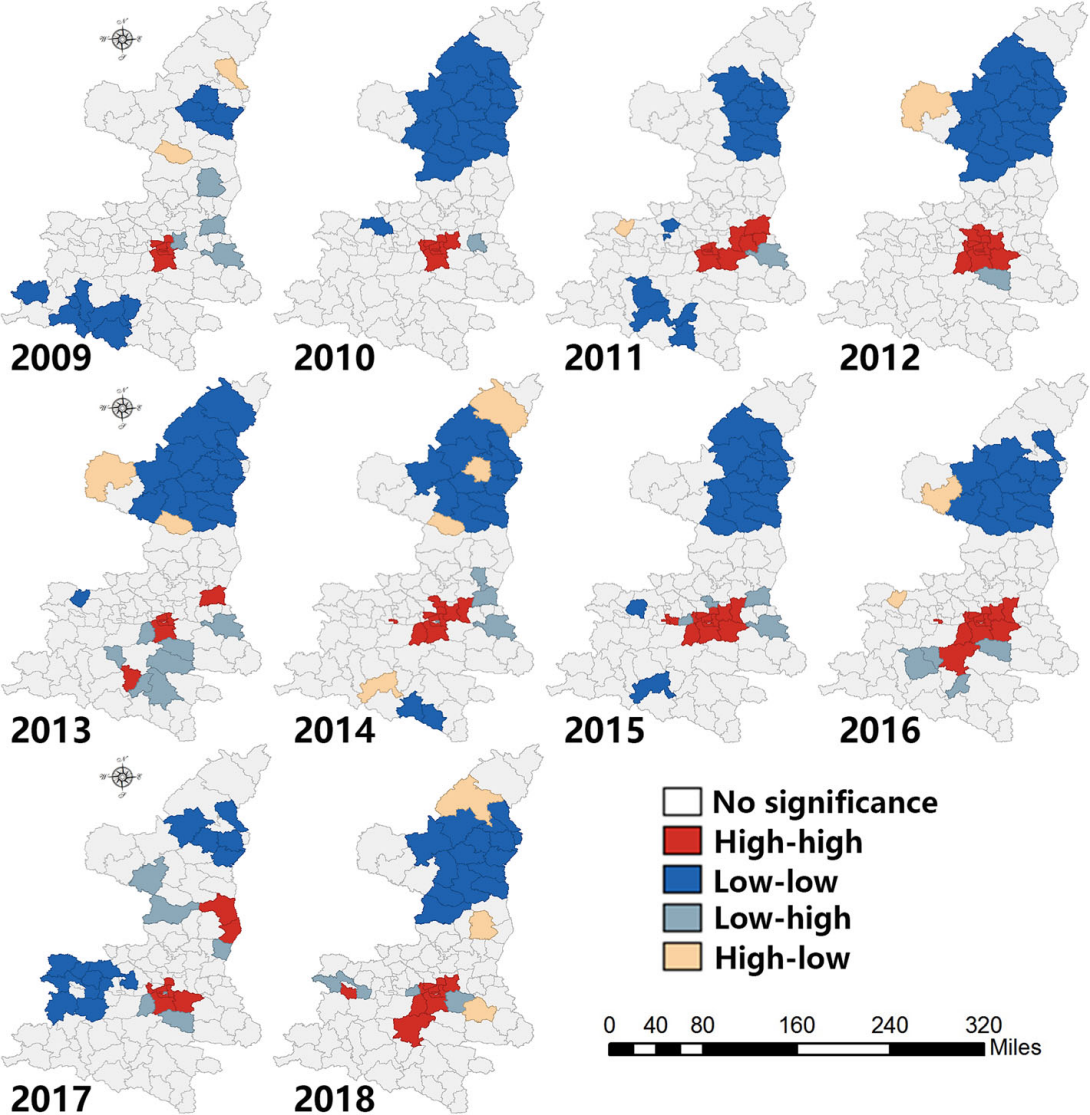
The spatial clusters of incidence rate of HFMD (made through GeoDa and ArcGIS, source of shape files: http://www.geodata.gov.cn/web/geo/index.html, under license without need for permission). *This figure was made by ourselves through GeoDa (Version 1.8.61, the University of Chicago, Chicago, IL, USA) and ArcGIS (Version 10.0, ESRI Inc., Redlands, CA, USA). The source of shape files was a public database, National Nature Resources and Geospatial basic information database of PRC (http://www.geodata.gov.cn/web/geo/index. html). Those shape files were under license without need for permission

Figure 4 shows the spatial cluster of morbidity of HFMD and reveals the geographical variation from 2009 to 2018. The HH cluster in central Shaanxi was witnessed throughout the entire research period (Xi’an, Xianyang and Weinan). Some southern regions adjacent to the central areas also showed evidence of the HH cluster. The counties susceptible to the HH cluster increased constantly. The agglomeration blocks were gradually extended to two or three. Conversely, the HL clusters were randomly scattered in Shaanxi province, mostly in the northern region. The counties included Jiaxian, Ganquan, Zizhou, Dingbian and other counties. The counties involved in the LH cluster were mainly located in the surrounding area of central Shaanxi. Regarding the LL cluster northern Shaanxi (Yan’an, Yulin) was involved. Western central Shaanxi (Baoji city) also experienced the LL cluster in 2017.

### Empirical results of spatial panel models

**Fig. 5:**
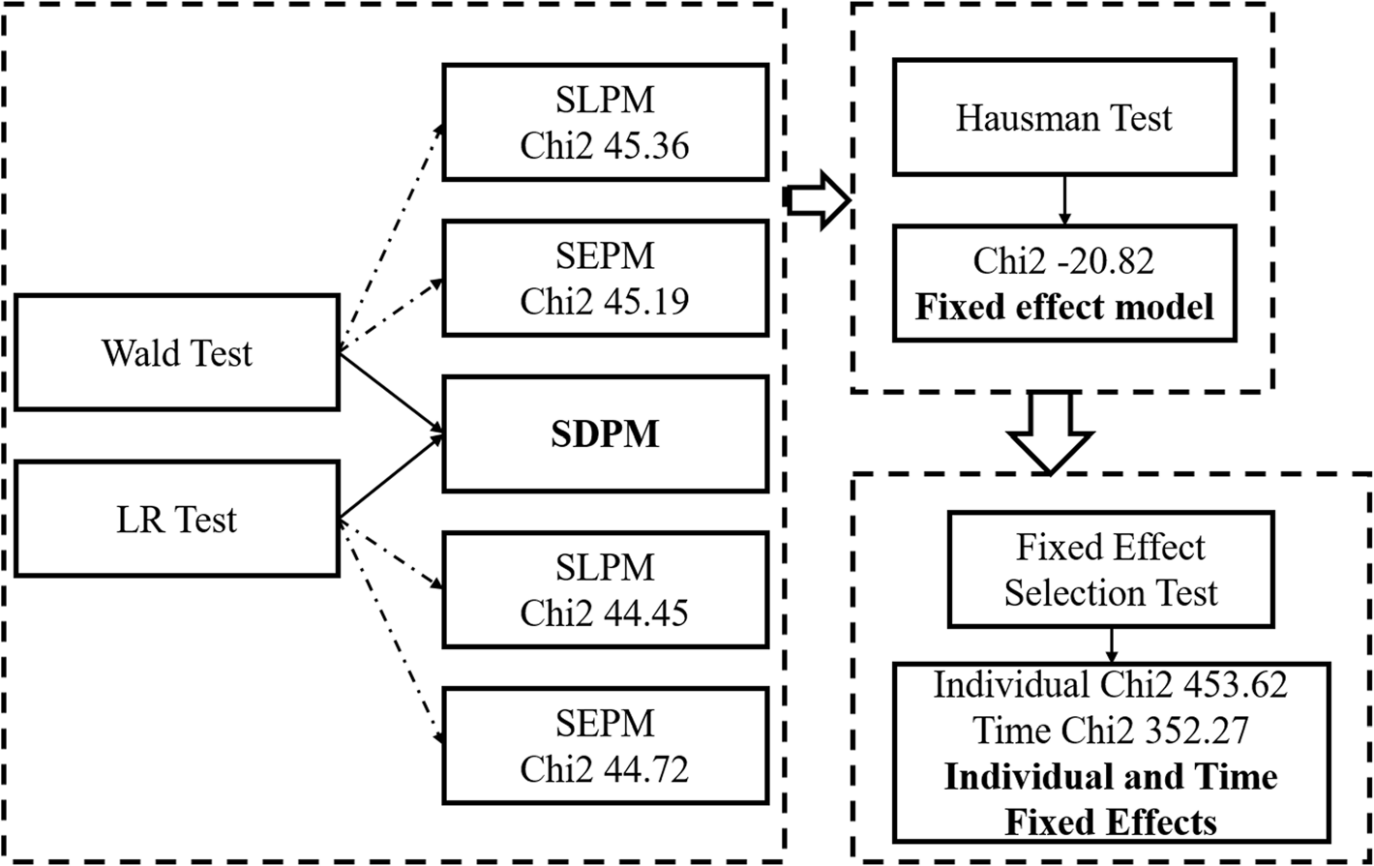
Model selection procedures of the spatial panel econometric models (made through Microsoft PowerPoint). *This figure was made by ourselves through Microsoft PowerPoint (Version 2013, Microsoft Crop, Redmond, WA, USA)

Table 6 displays all the estimation results of the spatial panel econometric models for HFMD. The model selection process is shown in Fig. 5 and demonstrated that the best model is SDPM, with individual- and time-based fixed effects. Among all three models, the application of SDPM requires multiple conditions, while SLPM and SEPM are simplified models of SDPM to some extent. Therefore, the first step requires the use of the Wald and LR tests to determine whether SDPM can be simplified to SLPM and SEPM (null hypothesis). In this study, all chi2 test results were significant; therefore, the null hypothesis was refused. The next step is to use the Hausman test to determine whether to use a fixed effect model or a random effect model. The fixed model was chosen if the chi2 test result was a negative number. Finally, time, individual or both fixed models were selected according to the test results.

**Table 6:** Estimation results of spatial panel econometric models for HFMD

Variable	SDPM with individual Fixed Effects	SDPM with Time Fixed Effects	SDPM with individual and Time Fixed Effects (Best model)	SDPM with Random Effects	SEPM with individual and Time Fixed Effects	SLPM with individual and Time Fixed Effects
Ln (waterfall)	0.115*** (3.79)	0.129*** (4.13)	0.064** (2.28)	0.119*** (3.90)	0.043 (1.48)	0.048 (1.68)
temperature	0.033 (0.71)	0.009 (0.59)	0.074 (1.87)	0.020 (0.49)	0.220*** (7.07)	0.216*** (6.82)
humidity	0.005 (0.48)	−0.021*** (−3.73)	0.005 (0.60)	0.001 (0.12)	0.021*** (2.90)	0.020*** (2.80)
W × ln (waterfall)	−0.110*** (−3.08)	− 0.206*** (− 3.78)	−0.224*** (−4.55)	−0.114*** (− 3.19)		
W × temperature	0.025 (0.53)	0.189*** (7.52)	0.047*** (3.12)	0.038 (0.93)		
W × humidity	− 0.004 (− 0.37)	0.079*** (6.36)	0.481*** (6.11)	− 0.000 (− 0.01)		
**ρ**	0.527*** (23.08)	−0.047 (−1.28)	− 0.082* (−2.23)	0.525*** (22.94)		− 0.050 (−1.36)
λ					−0.066 (−1.76)	
LLR	− 1947.0557	− 1821.5605	− 1673.4282	− 1968.8335	− 1698.6342	− 1699.2869
Rw^2^	0.3798	0.3555	0.3760	0.3792	0.3755	0.3755
Rb^2^	0.3354	0.4717	0.3557	0.3798	0.1764	0.1688
R^2^	0.3681	0.3593	0.3693	0.3682	0.3514	0.3502
Obs	1200	1200	1200	1200	1200	1200

Note: Standard error in parentheses, *** *p* < 0.01, **p < 0.05, * *p* < 0.1

According to the empirical results, the value **ρ** of the best model was significant and negative, which implied the existence of negative spillover spatial effects. The results of empirical results could not be interpreted directly. Thus, we analysed the incidence rate of HFMD and its determinants from direct and spill-over effects. The direct effects mean the influence from the determinants of local region such as the effect of meteorological factors in Xi’an to its incidence rate of HFMD, whereas the spillover effects reflect the influence from surrounding areas such as the effect of meteorological factors in Xi’an to incidence rate of HFMD in Weinan (an adjacent region).

### Decomposing the direct and spill-over effects

Tables 7 and 8 report the direct and spill-over effects independent of HFMD. Regarding the direct effects shown by the best model, rainfall had a positive association with the incidence rate of HFMD: with a 1% increases of rainfall in affected counties, the incidence rate of HFMD would increase 0.071%, while there was no significance when considering temperature and humidity.

**Table 7:** Direct effects of independent variables on HFMD

	SDPM with individual Fixed Effects	SDPM with Time Fixed Effects	SDPM with individual and Time Fixed Effects (Best model)	SDPM with Random Effects	SEPM with individual and Time Fixed Effects	SLPM with individual and Time Fixed Effects
Ln (waterfall)	0.106*** (3.61)	0.133***(4.14)	0.071** (2.43)	0.109***(3.65)	**–**	0.0493 (1.67)
temperature	0.040 (0.98)	0.006 (0.41)	0.066 (1.69)	0.028 (0.80)	**–**	0.215*** (6.98)
humidity	0.005 (0.58)	−0.021***(−3.95)	0.004 (0.43)	0.002 (0.21)	**–**	0.021*** (2.96)

Note: Standard error in parentheses, *** *p* < 0.01, **p < 0.05, * *p* < 0.1

**Table 8:** Spillover effects of independent variables on HFMD

	SDPM with individual Fixed Effects	SDPM with Time Fixed Effects	SDPM with individual and Time Fixed Effects (Best model)	SDPM with Random Effects	SEPM with individual and Time Fixed Effects	SLPM with individual and Time Fixed Effects
Ln (waterfall)	0.094 (1.86)	0.207***(3.00)	0.218*** (4.72)	0.098*(2.02)	**–**	0.002 (0.98)
temperature	0.082* (2.01)	0.182***(8.15)	0.450*** (6.19)	0.094**(0.013)	**–**	0.010 (1.34)
humidity	0.003 (0.25)	0.078***(6.41)	0.045*** (3.25)	0.000 (0.02)	**–**	0.001 (1.18)

Note: Standard error in parentheses, *** *p* < 0.01, **p < 0.05, * *p* < 0.1

Spill-over effects could be explained from two perspectives. When the coefficient was positive, the independent variables of one specific unit could positively affect the HFMD of surrounding counties; conversely, a negative coefficient would be interpreted as a negative association with the HMFD of the surrounding region. According to our analysis, all the independent variables displayed positive relationships with HFMD, which demonstrated that increases in rainfall, temperature and humidity in one county would increase the incidence rate of HFMD in adjacent counties. Regarding rainfall, a 1% increase would cause a 0.218% increase in the incidence rate of HFMD. With an increase in temperature of 1 °C, the incidence rate of HFMD of the surrounding counties would increase 0.450%. Considering humidity, a 0.045% incidence increase in the surrounding region could correspond to a 1% increase in humidity.

## Discussion

In recent years, HFMD has become an important public health problem worldwide. Since the 1990s, the disease has frequently broken out in the Asian-Pacific region. Large-scale HFMD outbreaks have occurred in China, with many severe cases and deaths. Shaanxi is a special province with three different climate zones; thus, it was chosen as the research area. In this article, we applied temporal and spatial analysis methods to display the characteristics of the HFMD incidence rate in Shaanxi at the county level from 2009 to 2018. Then, spatial econometric panel models were used to analyse the relationship between HFMD and meteorological factors. The evidence provided insights into potential solutions to diminish the disease incidence.

Based on the reported incidence rate of HFMD in Shaanxi from 2009 to 2018. The incidence rate of HFMD fluctuated during the research period. There was no distinct trend in the period of the investigation. In China, the targeted polices of HFMD had limited influence on the control and prevention of the disease. At the same time, the meteorological and socioeconomic factors changed during the research period. Meteorological factors such as temperature and humidity can directly affect the reproduction of pathogens and their survival time in the environment. Moreover, the children of migration accounted for seven-tenths of the total incidence of HFMD, at the same time, there was a predisposition associated with the condition of the systemic state of the adult, although it may appeared in immunocompetent adults. The immigration scale varied during the study period, which may could explain the fluctuation of incidence rate. Fang conducted a research about spatial characteristics of immigration of Shaanxi province, revealing that the immigration in central Shaanxi is HL cluster, which may could explain the situation of HFMD in that region [33]. Considering the seasonal trends, summer and autumn were the high-occurrence seasons, and the results were the same as the research result of Li [34], which was that the summer and autumn are the main outbreak seasons throughout China. The same results have also been found in Sichuan province [35], Shandong province [36], Guangxi province [37], Guangdong province [38], and Zhejiang province [39]. On one hand, in summer and autumn, the monsoon climate of China brings much precipitation, along with high temperature and high humidity, which could form a suitable environment for pathogen growth. On the other hand, the hot and humid environment can influence people’s lifestyle. For example, people are excessively depending on air conditioning to create a comfortable indoor environment now, but it could cause some health problems in a long run [40]. In general, as the highest season of HFMD incidence, summer should be paid more attention.

In consideration of HFMD, a spatial autocorrelation could be detected in Shaanxi according to the significant global Moran’s I. In fact, a spatial autocorrelation of HFMD has been found in other counties in southeast Asia, such as Thailand [41], Vietnam [42], and Malaysia [43]. Hot spots were witnessed in central Shaanxi (Guanzhong plain), which is a relatively rich area in Shaanxi. With the highly developed industrialization and the urban sprawl, the urban living environment in began to change. The large migrant population normally had a poor health lifestyle and poor knowledge of epidemic prevention measures. Thus, some high clusters were found on the border between Shaanxi and Shanxi. Cold spots were detected in northern Shaanxi (loess plateau). The northern region is relatively poor, with a population with low mobility compared with the central areas. At the same time, the meteorological environment was not suitable for the growth of pathogens. The humidity, temperature and rainfall were relatively low in contrast with other regions in Shaanxi. Considering the HL cluster, there were several interesting characteristics. On one hand, high and low clusters could be witnessed at the same time within specific municipal cities. The core area would be normally surrounded by low clusters. In general, different clusters could be detected inside and outside municipal cities due to different socio-economic or meteorological factors. On the other hand, some border areas of the province showed high clusters compared with the surrounding region, such as Dingbian. The county is located in the junction of four provinces: Shaanxi, Gansu, Ningxia and Neimenggu. Thus, the region was easily effected by the policy, economic or meteorological factors of the adjacent provinces. LH clusters were mainly found in surrounding areas of high cluster regions. In addition, some southern regions in Shaanxi displayed low clusters in comparison with the central region. The southern region is characterized by low latitude, which means abundant precipitation, with a humid climate and high temperature.

Regarding the meteorological factors analysed in this study, rainfall, temperature and humidity were positively associated with the incidence rate of HFMD in specific regions. Du [44], Zheng [45], Wu [46], and Zhang [47] reported the same results in some high-risk areas such as Huanan, Hainan and Guangdong. In this study, we found a relationship between the meteorological factors of surrounding regions and the incidence rate of one specific region. The rainfall, temperature and humidity of surrounding regions were also positively associated with the incidence rate of HFMD of local counties. Naturally, the adjacent region normally experiencing same meteorological situation.

The strengths of this study are as follows: this study analysed the temporal and spatial analysis of HFMD in Shaanxi, China, and was the first study to analyse the situation of Shaanxi in the context of other counties, and the influence on HFMD from meteorological factors in the surrounding regions were explored for the first time. Moreover, the visualization of diseases at the county level provides a systematic and comprehensive method to understand the changing patterns of HFMD in Shaanxi. However, there were also some limitations in this study. In China, the meteorological data are obtained from monitoring points that are distributed in different regions. Thus, the meteorological data were not very precise. Furthermore, other determinants should be incorporated in future research.

## Conclusion

In conclusion, this study aimed to analyse HFMD outbreaks and their spatial-temporal patterns in Shaanxi province, China. The incidence rate of HFMD displayed no trend from a temporal perspective. A high incidence rate of HFMD was observed from June to September, a season characterized by heavy rain, high temperature, and high humidity. The high-incidence spots were mainly located in rich regions.

Based on the findings in our study, spatial-temporal analysis and its tools proved to be effective research method for analysing the outbreak of HFMD, especially for local governments. The results could be applied by governments and the general public to take effective measures to prevent disease. From the government perspective, the region-targeted policies could be enacted and implemented in the future according to specific situations of different areas and their meteorological determinants. At the same time, meteorological conditions normally extend to wide-ranging regions; thus, cooperation between surrounding regions is necessary. Furthermore, the local governments need take responsibility for the health management of immigrants and mobile populations. From the research institution perspective, spatial-temporal analysis should applied in the research of HFMD in central and local regions. From the citizen perspective, lifestyle could influence the control and prevention of HFMD; thus, citizens should cultivate good living habits, such as hand-washing and sanitary eating habits. In addition, citizens need to improve their awareness and knowledge of disease prevention.

## Supplementary Information

**Additional file 1.**

**Additional file 2.**

**Additional file 3.**

## Data Availability

The datasets of incidence rate of HFMD that support the findings of this study are available from Shaanxi Provincial Centre of Disease Control and Prevention but restrictions apply to the availability of these data, which were used under license for the current study, and so are not publicly available. Data are however available from the authors upon reasonable request and with permission of Shaanxi Provincial Centre of Disease Control and Prevention. The meteorological data generated or analyzed during this study are included in this published article (Appendix 1).

## Abbreviations

HFMD: Hand-foot-mouth disease
WHO: World Health Organization
EV71: Enterovirus 71
CDC: Chinese Center for Disease Control and Prevention
BTT: Beijing-Tianjin-Tangshan
GAM: General additive model
SXCDC: Shaanxi Provincial Center for Disease Control and Prevention
CMDC: China Meteorological Data Service Center
CMA: China Meteorological Administration
HH: High-high
HL: High-low
LH: Low-high
LL: Low-low
SLPM: Spatial Lag Panel Model
SEPM: Spatial Error Panel Model
SDPM: Spatial Durbin Panel Model
USA: The United States
WA: Washington
IL: Illinois
TX: Texas
CA: California
LISA: Local indicators of spatial association
ESRI: Environmental Systems Research Institute
Obs: Observations
Std. Dev.: Standard deviation
Min.: Minimum
Max: Maximum
IN: Incidence rate
LLR: Log Likelihood Ratio
