# Supplementary material for: Spatiotemporal characteristics and meteorological determinants of hand, foot and mouth disease in Shaanxi Province, China: a county-level analysis
Source: BMC Public Health. 2021 Feb 17;21:374. doi: 10.1186/s12889-021-10385-9 (PMC7890844; doi:10.1186/s12889-021-10385-9)
Supplement: Supplementary file 2 — Additional file 2. [file 12889_2021_10385_MOESM2_ESM.pdf]

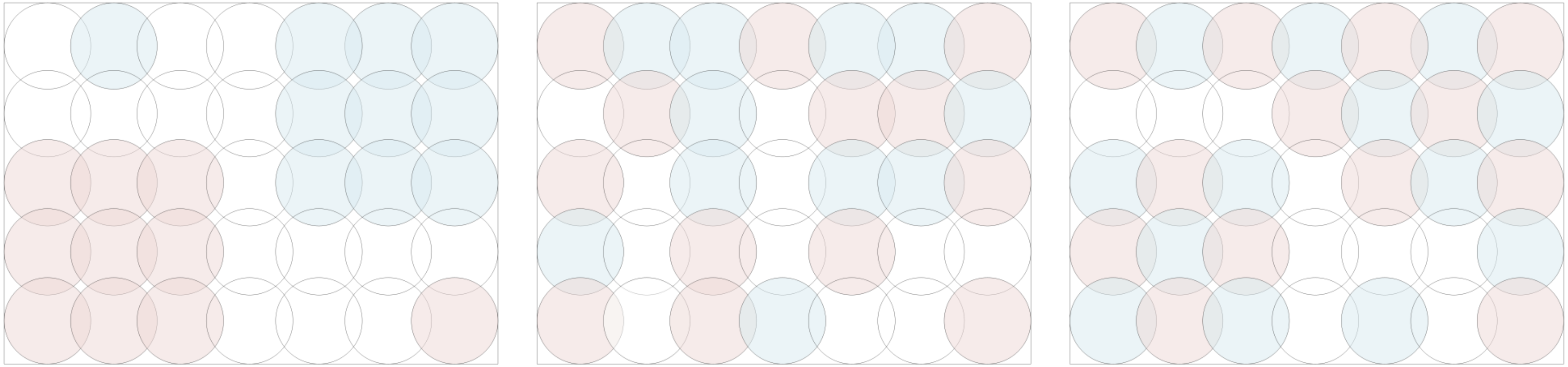

Appendix 2 Demonstration of different type spatial cluster (made through Microsoft PowerPoint, this figure does not need any other data)

*This figure was made by ourselves through Microsoft PowerPoint (Version 2013, Microsoft Crop, Redmond, WA, USA).
