# Supplementary material for: Spatiotemporal characteristics and meteorological determinants of hand, foot and mouth disease in Shaanxi Province, China: a county-level analysis
Source: BMC Public Health. 2021 Feb 17;21:374. doi: 10.1186/s12889-021-10385-9 (PMC7890844; doi:10.1186/s12889-021-10385-9)
Supplement: Supplementary file 3 — Additional file 3. [file 12889_2021_10385_MOESM3_ESM.docx]

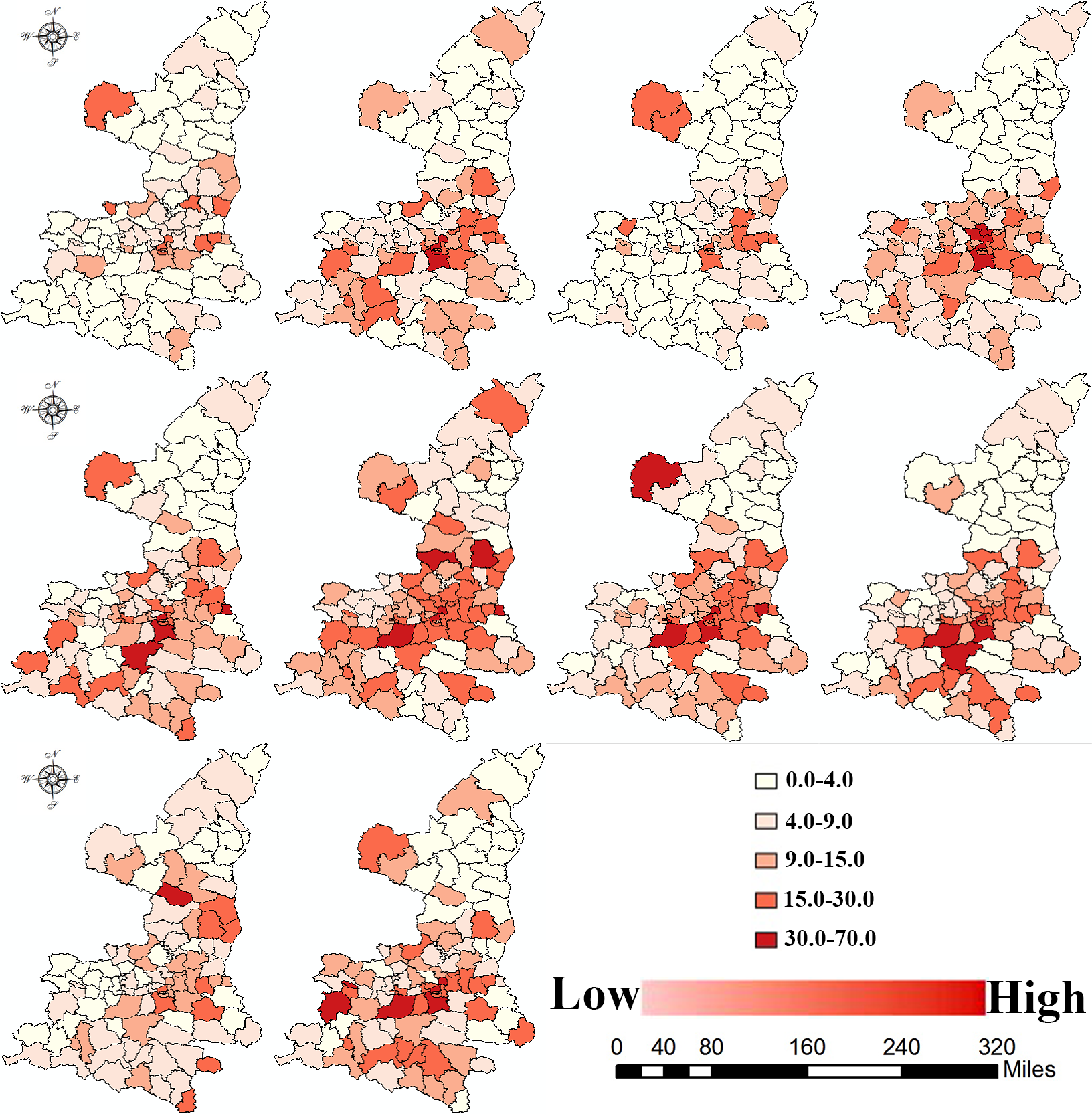


Appendix 3 Map showing the hierarchy of the incidence rates for HFMD

*This figure was made by ourselves through GeoDa (Version 1.8.61, the University of Chicago, Chicago, IL, USA) and ArcGIS (Version 10.0, ESRI Inc., Redlands, CA, USA). The source of shape files was a public database, National Nature Resources and Geospatial basic information database of PRC (http://www.geodata.gov.cn/web/geo/index. html). Those shape files were under license without need for permission.
